# Bioactive Amento flavone isolated from *Cassia fistula* L. leaves exhibits therapeutic efficacy

**DOI:** 10.1007/s13205-017-0599-7

**Published:** 2017-04-11

**Authors:** M. Srividhya, H. Hridya, V. Shanthi, K. Ramanathan

**Affiliations:** 1 Department of Biotechnology, School of Bio Sciences and Technology, VIT University, Vellore, Tamilnadu 632014 India; 2 Karpaga Vinayaga College of Engineering and Technology, Chinnakolambakkam, Kanchipuram Dt, Madhurantagam, India

**Keywords:** *Cassia fistula* L., Amentoflavone, Biflavonoid, Antioxidant, Cytotoxicity

## Abstract

Novel natural compounds endowed with sound bioactivities are currently the utmost need as leads toward drug discovery. For the first time, here, we report the presence of Amentoflavone (biflavonoid) in the leaves of *Cassia fistula* L. Structural characterization was carried out using ultraviolet–visible spectrophotometer, Fourier transform infrared, nuclear magnetic resonance, and thin-layer chromatography. The isolated compound was further evaluated for its bioactivity. The compound demonstrated moderate cytotoxicity in liver carcinoma (HepG2) cells, and the comparative analysis for the standard and normal compound has also been validated. Antioxidant potential was assessed by DPPH assay. Furthermore, efficacy of the compound in the aforesaid assays asserts its bioactivity and subsequently its importance as a potent therapeutic. Our study strongly suggests that Amentoflavone present in the leaf extracts of *C. fistula* L. definitely holds promise in the pharmaceutical industry.

## Introduction

The use of plants and their constituents in primary health care has ancient history as old as human beings. Various medicinal plants have proven therapeutics implication in the health management via antioxidant, anti-inflammatory, anti-diabetic, and other biological activities (Rahmani et al. 2014a, 2014b; Rahmani and Aly 2015). *Cassia fistula* L. (Caesalpiniaceae) is commonly called Indian Laburnum and is native to India, Amazon, Sri Lanka, and extensively available in various countries, including Mauritius, South Africa, Mexico, China, West Indies, East Africa, and Brazil (Bahorun et al. 2005; Rajagopal et al. 2013). The main medicinal property of *C. fistula* L. is mild laxative suitable for children and pregnant women. It is also used as a purgative due to the wax aloin and a tonic and has been reported to treat many intestinal disorders, such as ulcers (Bhalodia et al. 2012). In the Indian literature, this plant has been described to be useful against skin diseases, liver troubles, tuberculosis glands, and its use in the treatment of hematemesis, pruritus, leucoderma, and diabetes and has also been suggested that *C. fistula h*as anti-microbial and anthelminthic properties (Vasudevan et al. 2009; Panda et al. 2011; Siva 2007) and also possesses significant hepatoprotective activity (Das et al. 2008). *C. fistula* extract is used as an anti- periodic and anti-rheumatism, and the leaf extract is also indicated for its antitussive and wound healing properties (Senthil kumar et al. 2006). The several species of Cassia are known to produce a variety of phenolic metabolites with novel structures, especially flavonoids. Therefore, the objective of the study was to investigate the novel compound and their bioactivity in leaves of *C. fistula.* For the first time, a novel Amento flavonone has been identified in the leaves of *C. fistula* by systematic bioassay-guided fractionation of the ethanolic extract. The therapeutic efficacy of Amento flavonone has been reported in this paper through DPPH and cytotoxicity to HepG2 (human hepatic carcinoma) cell line. The present study explores the role of the novel compound obtained from the leaves ethanolic extract of *C. fistula*. To the best of our knowledge, this is the first report on Amento flavonone from *Cassia fistula* L.

## Materials and methods

### Plant material

Leaves of *C. fistula* Linn. were collected from Madhurantagam village, Kanchipuram District, Tamil Nadu, India and subsequently maintained as a herbarium sample with a voucher number PARC/2015/3071. The samples were cleansed thoroughly in water, air dried and ground into semi-granulated powder to be used for further processing.

### Preparation of extract and isolation

The fresh leaves about 60 g was subjected to exhaustive extraction in a soxhlet apparatus using 100% ethanol (250 ml) as a solvent. This extracts, referred to as the crude extract here onwards, yielded an extract of 2.5 g. Thereafter, 1 g of dried ethanolic crude extract was subjected to column chromatography with 30 g silica gel (60–120 mesh). In column, hexane with silica gel was poured into the column and gradually added slurry. The fraction was eluted from the crude extract with hexane and ethyl acetate (4:1). Fractions obtained from column chromatography were checked individually, and identical fractions were pooled together based upon the TLC band. This process continued until we obtain minimum compound with maximum purity.

### Phytochemical characterization

The yielded compound is semisolid (20 mg), and the absorbance wavelength of isolated molecules was analyzed using UV–Vis spectrophotometer (Systronics AU-2401 UV–Vis double beam spectrophotometer). The spectral values of the isolated compound were identified with the peak obtained in the spectral range of 250–400 nm. FTIR analysis was carried out using a Shimadzu IR Affinity-1 Fourier transform infrared spectrophotometer. Spectra were collected and treated using the OMNIC software. ^1^H and ^13^C NMR spectra were determined in CDCl_3_ solution at 400 MHz using Bruker Ascend Model. All spectra were recorded on a Bruker AC-250 spectrometer at 5.87 T (250.133 and 62.896 MHz). The compound was dissolved in CD3OD (99.8% D) and estimated as per the standard protocol. Amentoflavone present in the sample was identified by matching the retention time against those of the standard, and the content of Amentoflavone was determined using calibration curve. The data analysis was done using the Empower 2 software.

### DPPH scavenging activity

The ability of the isolate to scavenge free radicals was confirmed by estimation of its DPPH radical scavenging activity, as described by Ohinishi et al. (1994). Five different concentrations (6, 12, 25, 50, and 100 µg/ml) of the pure compound were prepared. To each, 3 ml of DPPH (0.1 mM/ml in ethanol) solution was added. The entire reaction mixture was incubated in complete darkness for 30 min, and thereafter, its absorbance was read at 517 nm. The results were compared with ascorbic acid, which is a standard antioxidant equivalent. Percentage radical scavenging (%RS) activity was calculated using the formula:

$$\% {\text{RS }} = \, \left( {\left[ {A_{\text{br}} - \, A_{\text{ar}} } \right]/A_{\text{br}} } \right) \, \times \, 100$$

where *A*_br_ is the absorbance before reaction and *A*_ar_ is the absorbance after the reaction has taken place. The results of the comparative analysis of the standard and Amentoflavone were shown in Fig. 3.

### Cytotoxicity by MTT assay

HepG2 cells were cultured in Dulbecco’s modified medium (DMEM) supplemented with glutamine (0.6 g/l), gentamicin (25 mg/ml), and 10% fetal calf serum at 37 °C and humidified with 5% CO_2_. Cells were plated in a 96-well plate (10^5^ cells/well for adherent cells or 0.3 × 10^6^ cells/well for suspended cells in 100 µl of medium). After 24 h, the standard and compound (6, 12, 25, 50, and 100 µg/ml) dissolved in DMSO (1%) was added to each well and incubated for 96 h. At the end of 96 h incubation, the medium in each well was replaced with fresh medium containing 0.5 mg/ml of MTT. The growth of HepG2 cells was quantified by the ability of living cells to reduce the yellow dye MTT to a blue formazan product. Four hours later, the formazan product of MTT reduction was dissolved in DMSO and absorbance was measured at 570 nm (Ferrari et al. 1990). The change in color indicates the cell viability and toxicity. The absorbance was noted down at 570 nm, and the % cell survival was calculated using the following formula:

$$\% {\text{ Cell survival }} = \, \left( {{\text{OD}}_{\text{T}} /{\text{OD}}_{\text{C}} } \right) \, \times { 1}00.$$

OD_T_ and OD_C_ are the mean absorbance of the treated and the control cells, respectively. The concentration of the extract that caused a half-maximal inhibition of cell proliferation (IC_50_) was determined obtained from a semi-log plot of the concentrations against the percentage of cell survival.

## Results and discussion

**Fig. 1:**
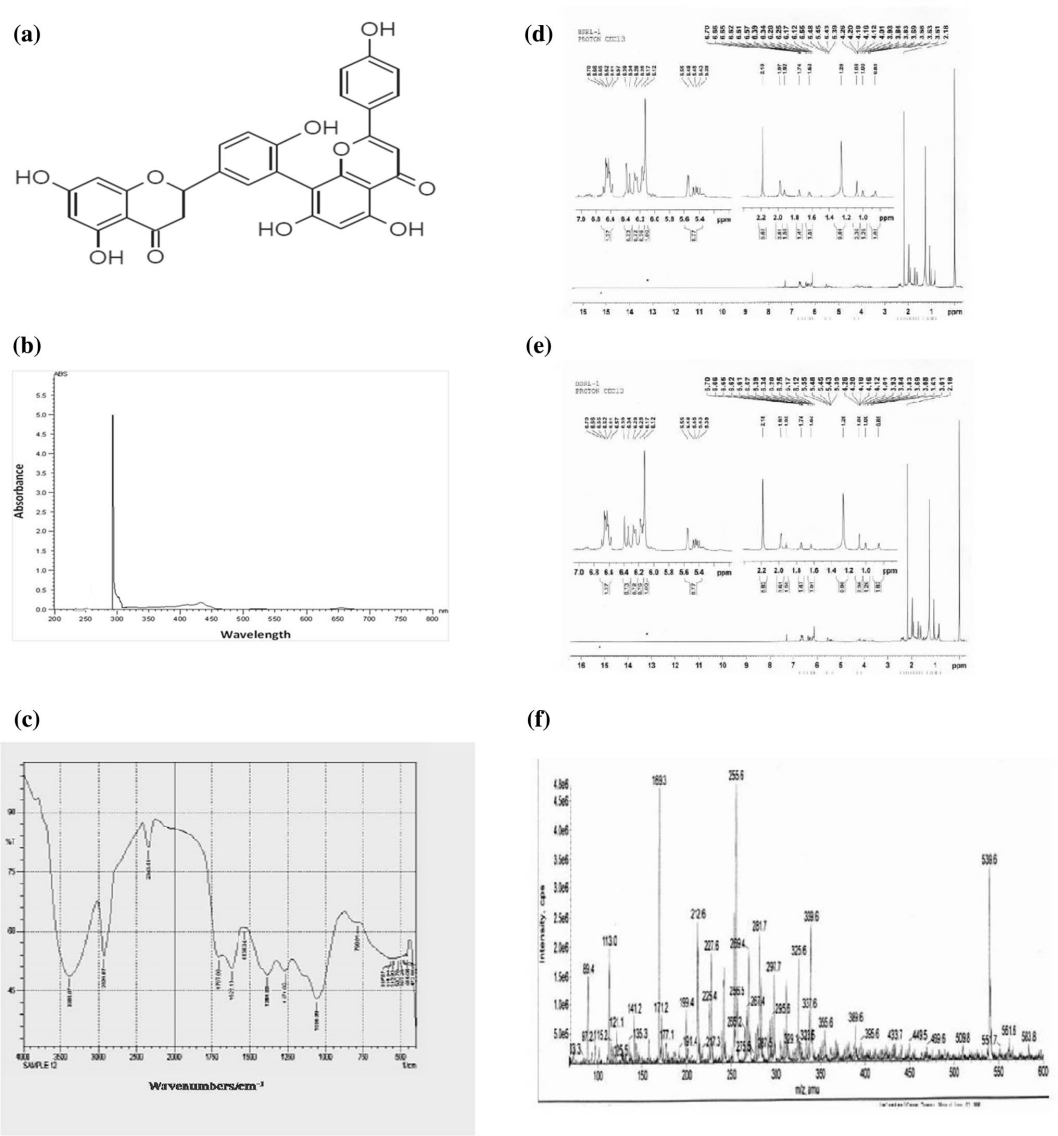
**a** Structure of Amentoflavone (biflavonoid) elucidated by NMR; **b** UV visible spectrum of Amentoflavone extracted from the ethanolic extracts of *Cassis fistula* L.; **c** FTIR spectrum of isolated Amentoflavone from the ethanolic leaf extract of *Cassia fistula L*. in the 4000–400 cm^−1^ region; **d** Structure elucidation by C^13^NMR spectra of isolated compound; **e** Structure elucidation by NMR for isolated compound (proton donor atom); and **f** Structure elucidation by NMR spectra for isolated compound (MS)

**Fig. 2:**
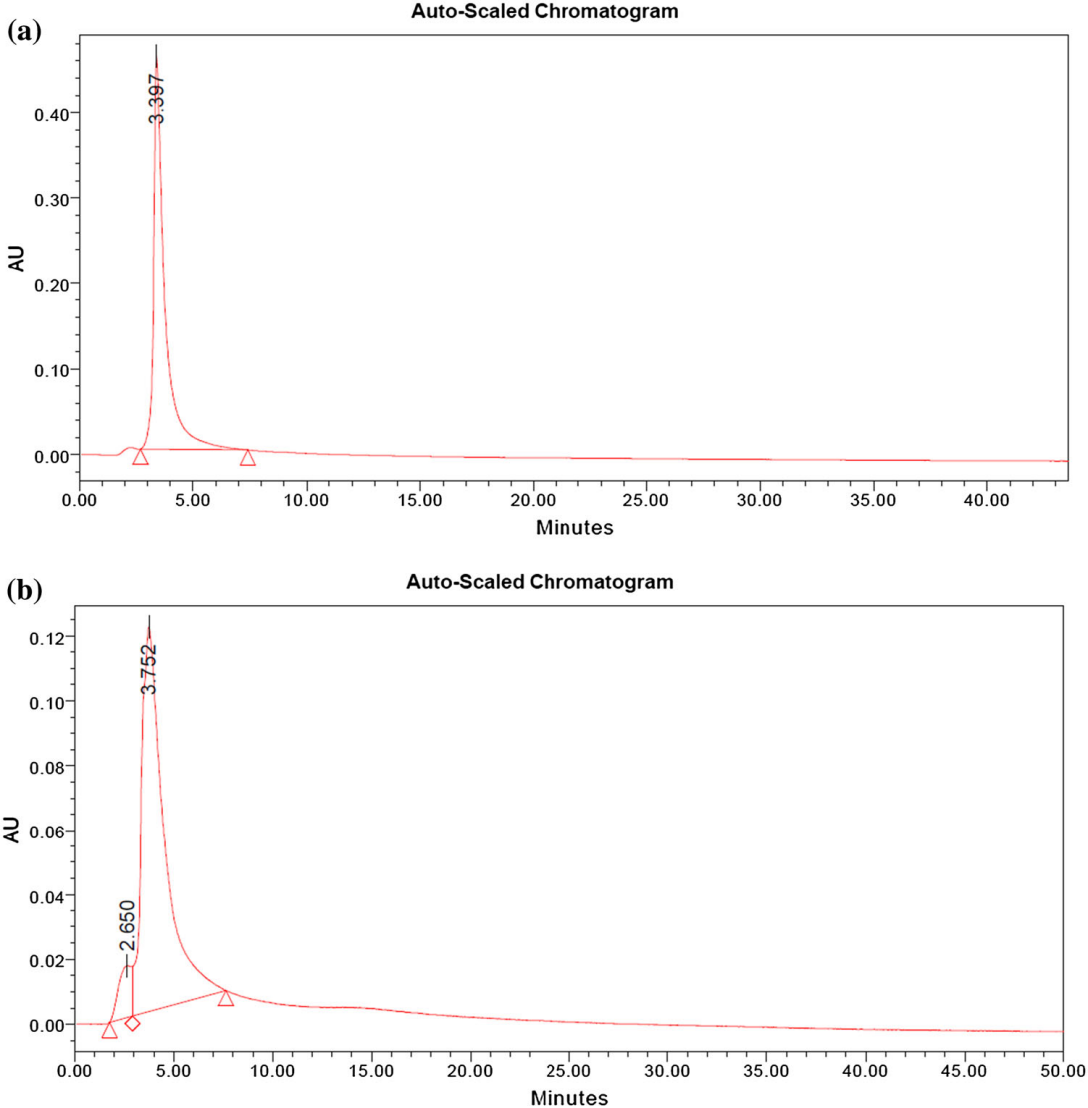
HPLC data of **a** standard and **b** isolated Amentoflavone

**Fig. 3:**
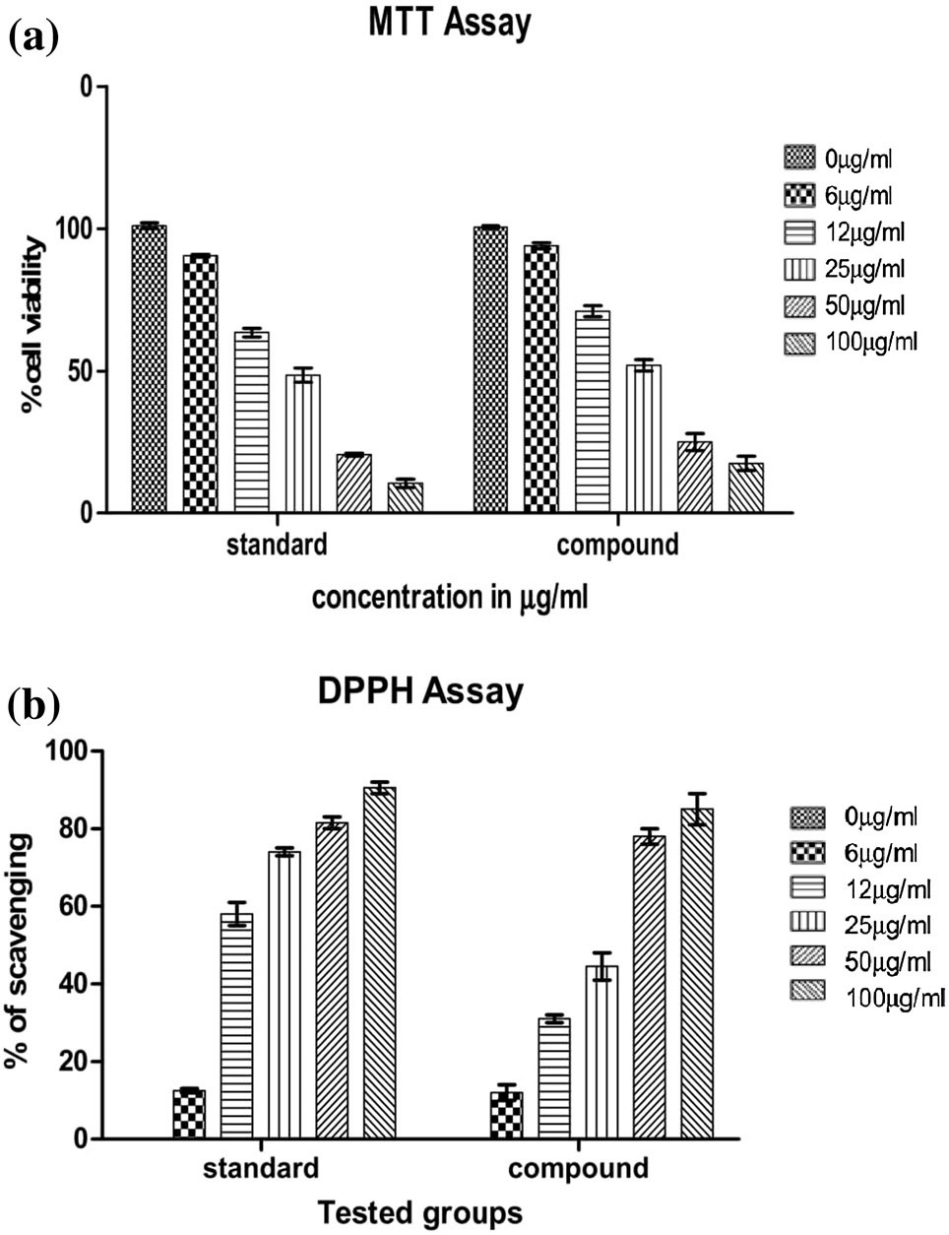
Comparative study of the cytotoxicity and antioxidant activity of the novel and standard Amentoflavone by **a** DPPH and **b** MTT assay

For the foremost time, the present research investigation afforded Amentoflavone, from the leaves of *Cassia fistula*. The yielded Amento flavonone is semisolid (20 mg), and the characterization of the isolated molecules was analyzed using multispectroscopic techniques, such as UV–Vis, FTIR, and NMR (Fig. 1). Further HPLC analysis was carried out, Amento flavonone present in the sample was identified by matching the retention time against those of standard, and the content of Amentoflavone was determined using calibration curve. The data analysis was done using the Empower 2 software, and the results of the standard and novel compound are shown in Fig. 2. Thus, for the first time, we report the presence of Amentoflavone in *Cassia fistula* leaves. Literature review showed Amentoflavone to be a potential bioactive compound. Amentoflavone is an irreversible inhibitor of lymphocyte proliferation (Guruvayoorappan and Kuttan 2008), an inhibitor of phospholipase, and an inhibitor of nitric oxide synthase in macrophages (Woo et al. 2005). Leaves of the species appear to be potential sources for Amento flavone. They can be extracted in sufficient amounts from these species, as they are prevalent in most of the places. Reactive oxygen species and free radicals are unstable, highly reactive molecules generated during normal biochemical metabolism and pathological conditions which damage biological macromolecules, such as DNA, protein, lipids ultimately leading to apoptosis (Francis et al. 2010, Jayaprakasha et al. 2006). Intake of antioxidants plays an important role in body’s defense mechanism to defend against free radicals and ROS (Rimm et al. 1993). Destroying activity of free radicals is sheltered by antioxidant by scavenging (Siva et al. 2011). The hunt for safe and potent phyto-therapeutic with antioxidant property is on high demand (Bhakta and Siva 2012). Furthermore, the therapeutic efficacy of the Amentoflavone was studied by DPPH and cytotoxic studies (Fig. 3). Dose-dependent antioxidant and cytotoxic activity was exhibited by Amentoflavone. The IC_50_ value of Amentoflavone was determined to be 29.7 µg/ml. Thus, Amentoflavone inactivates the singlet oxygen and scavenge the free radicals. The cytotoxicity of HepG2 cells upon Amentoflavone treatment was observed. Dose-dependent cytotoxicity was observed with an IC_50_ value of 25.3 µg/ml. Thus, the potent therapeutic efficacy of Amentoflavone is revealed.

## Conclusion

Hence, for the foremost time, it has been reported that *C. fistula* L. can be considered as a potential source for Amentoflavone. The findings from this work revealed the provided proof for the functional value of the medicinal potential of *Cassia fistula* L. However, further study should be carried out to determine the possible mechanisms of action.
